# Urothelial Papilloma of the Urinary Bladder in a Child

**DOI:** 10.1002/ccr3.71405

**Published:** 2025-10-30

**Authors:** Sogol Alesaeidi, Samin Alavi, Ahmad Khaleghnejad Tabari, Maryam Kazemi Aghdam, Ashraf Kheiri

**Affiliations:** ^1^ Pediatric Congenital Hematologic Disorders Research Center, Research Institute for Children's Health Shahid Beheshti University of Medical Sciences Tehran Iran; ^2^ Pediatric Surgery Research Center Research Institute for Children's Health, Shahid Beheshti University of Medical Sciences Tehran Iran; ^3^ Pediatric Pathology Research Center Shahid Beheshti University of Medical Sciences Tehran Iran

**Keywords:** children, hematuria, urinary bladder, urothelial papilloma

## Abstract

Urothelial papilloma (UP) of the bladder is an extremely rare benign neoplasm in pediatrics, often manifested as gross hematuria. These lesions are typically small, well‐circumscribed, located on the lateral or posterior bladder wall, frequently near the ureteral orifice. These small masses typically have a good prognosis with a favorable outcome.

## Graphical abstract

An exophytic mass located approximately 1 cm away from the left ureteral orifice on the left lateral bladder wall.

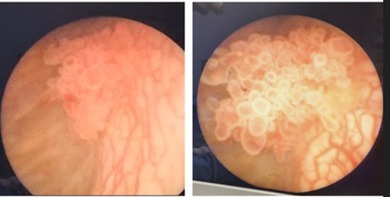

## 1 Case Presentation

**FIGURE 1:**
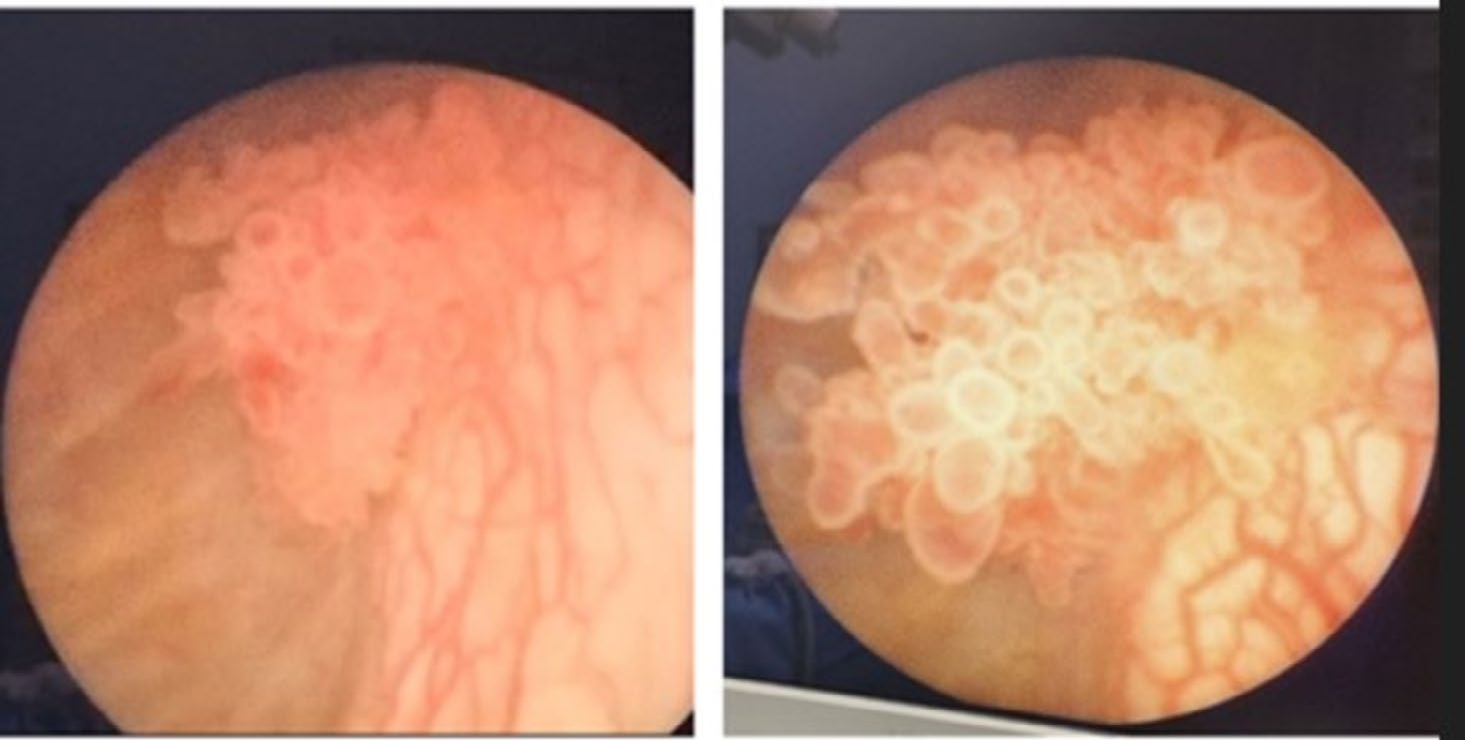
Cystoscopic view of the bladder papilloma showing papillary projections. The image shows an exophytic mass located at the left lateral bladder wall.

**FIGURE 2:**
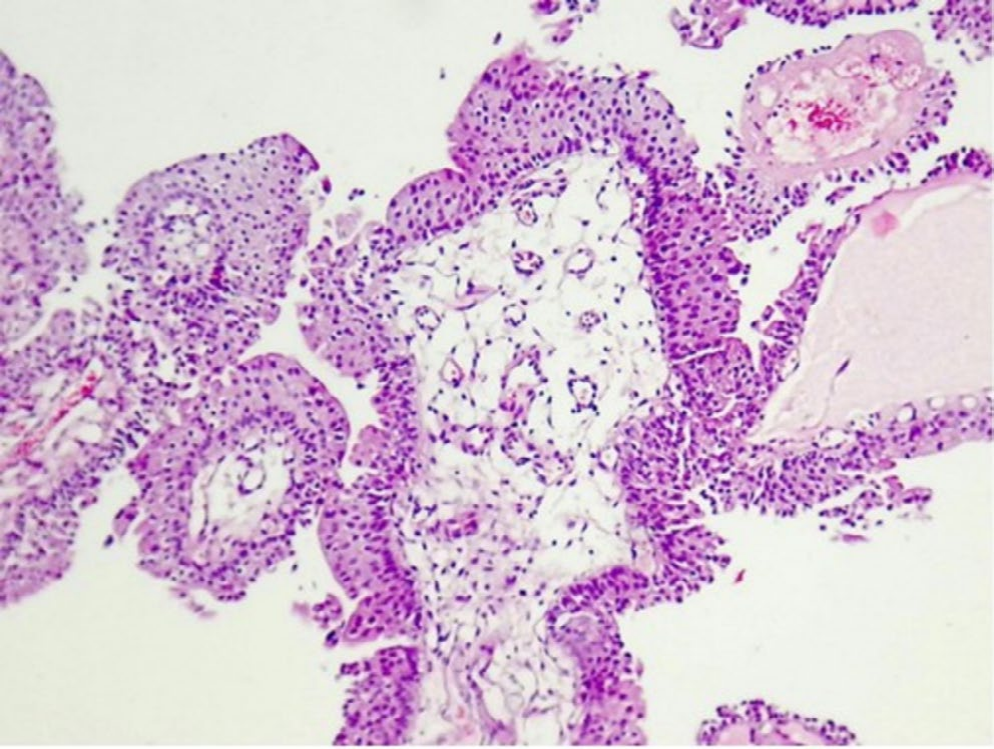
Papillary structures with edematous stroma, lined by bland‐looking urothelial cells (H&E ×200).

**FIGURE 3:**
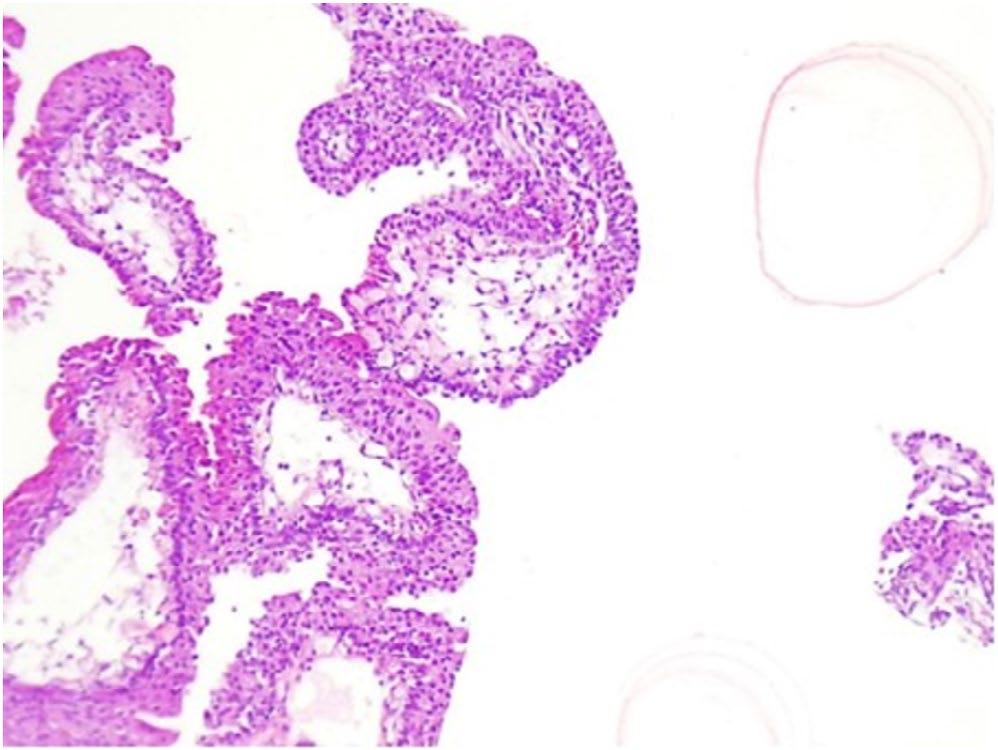
Histopathological section highlighting the normal urothelial lining of the papillary structures (H&E ×200).

An 8‐year‐old male, the first child of non‐consanguineous parents, presented with a one‐year history of intermittent terminal hematuria. The past medical and family history was unremarkable. Physical examination was non‐contributory. Given the persistence of hematuria, an abdominal ultrasound was performed which identified a hyperechoic mass measuring 7 × 14 mm with an internal vascular network located on the left lateral bladder wall. Both kidneys appeared normal, with no evidence of hydronephrosis or other abnormalities. Subsequently, cystoscopy revealed a trabeculated bladder containing an exophytic mass measuring approximately 5 × 10 mm located approximately 1 cm away from the left ureteral orifice on the left lateral bladder wall (Figure 1). The surface of the lesion appeared smooth with no evidence of ulceration or necrosis. The surrounding bladder mucosa was intact without significant inflammation or trabeculation. Microscopic examination of the resected mass by cystoscopy revealed urothelial tissue with distinct papillary structures and slender fibrovascular cores, without evidence of fusion. The urothelium, lining the papillary fronds appeared normal with no atypical features or increased mitotic activity. A prominent umbrella cell layer was observed on the surface of the urothelium. Based on histopathological findings, the lesion was diagnosed as a urothelial papilloma (Figures 2 and 3).

The postoperative follow‐up included a non‐contrast abdominopelvic CT scan, which showed no indications of local recurrence or involvement of adjacent organs. Over the course of a one‐year follow‐up, there were no observed signs of tumor recurrence or evidence of distant metastasis.

## 2 Discussion

UP is a benign exophytic lesion most commonly localized in the posterior or lateral wall of the urinary bladder. According to some studies, UP accounts for approximately 13% of bladder lesions in children and adolescents (ages 4–20 years) and 7.7% in adults. The distinction between UP and other papillary urothelial lesions is essential for accurate diagnosis and appropriate management [1, 2]. Cystoscopy and transurethral resection of the lesion appear to be the best therapeutic approach in the majority of cases to determine a small mass located on the lateral or posterior wall of the bladder near the ureteric orifices [3].

Histologically, bladder papilloma consists of delicate fibrovascular cores lined by normal urothelial cells without significant atypia or mitotic activity. Although the prognosis for bladder UP is generally excellent, there is a small risk of recurrence or progression to low‐grade papillary urothelial carcinoma, especially in cases with multifocal or recurrent tumors [1]. The diagnosis of urothelial papilloma is primarily based on light microscopy, enabling detailed assessment of its architectural and cytological features. While immunohistochemistry may aid in pathologic diagnosis, it is unnecessary for identifying this benign lesion.

Bladder papilloma should be considered in the differential diagnosis of unexplained hematuria in children. Given the low but present risk of recurrence and the potential for delayed complications, long‐term follow‐up remains essential.

## Author Contributions

**Sogol Alesaeidi:** data curation, resources, writing – original draft. **Samin Alavi:** investigation, supervision, writing – review and editing. **Ahmad Khaleghnejad Tabari:** validation, visualization. **Maryam Kazemi Aghdam:** methodology, visualization. **Ashraf Kheiri:** writing – original draft.

## Consent

A written informed consent was obtained from the patient to publish this report in accordance with the journal's patient consent policy.

## Conflicts of Interest

The authors declare no conflicts of interest.

## Data Availability

All the required data exists in the manuscript. The original imaging can be provided by the authors upon request.
